# Identification of natural products and FDA-approved drugs for targeting cancer stem cell (CSC) propagation

**DOI:** 10.18632/aging.204412

**Published:** 2022-12-01

**Authors:** Gloria Bonuccelli, Federica Sotgia, Michael P. Lisanti

**Affiliations:** 1 Translational Medicine, School of Science, Engineering and Environment, Biomedical Research Centre, University of Salford, Greater Manchester, United Kingdom

**Keywords:** cancer stem cells, drug screening, natural compounds, FDA approved drugs, mammospheres

## Abstract

Here, we report the identification of key compounds that effectively inhibit the anchorage-independent growth and propagation of cancer stem cells (CSCs), as determined via screening using MCF7 cells, a human breast adenocarcinoma cell line. More specifically, we employed the mammosphere assay as an experimental format, which involves the generation of 3D spheroid cultures, using low-attachment plates. These positive hit compounds can be divided into 5 categories: 1) dietary supplements (quercetin and glucosamine); 2) FDA-approved drugs (carvedilol and ciprofloxacin); 3) natural products (aloe emodin, aloin, tannic acid, chlorophyllin copper salt, azelaic acid and adipic acid); 4) flavours (citral and limonene); and 5) vitamins (nicotinamide and nicotinic acid). In addition, for the compounds quercetin, glucosamine and carvedilol, we further assessed their metabolic action, using the Seahorse to conduct metabolic flux analysis. Our results indicate that these treatments can affect glycolytic flux and suppress oxidative mitochondrial metabolism (OXPHOS). Therefore, quercetin, glucosamine and carvedilol can reprogram the metabolic phenotype of breast cancer cells. Despite having diverse chemical structures, these compounds all interfere with mitochondrial metabolism. As these compounds halt CSCs propagation, ultimately, they may have therapeutic potential.

## INTRODUCTION

Cancer stem-like cells (CSCs) represent a small sub-population of cancer cells [1] (< 1%) that are clinically responsible for resistance to radiotherapy and chemotherapy, the development of tumour recurrence, and the formation of metastases [2–4]. Key distinguishing features of CSCs are pluripotency, self-renewal and the ability to undergo anchorage-independent growth, favouring their propagation and metastasis [5, 6]. Unfortunately, conventional therapies are frequently not able to eradicate CSCs. For this reason, there is a clinical urgency to intervene via the discovery of new drugs that can inhibit the propagation of cancer stem cells, that can perhaps be used in conjunction with more traditional therapies.

CSCs show metabolic plasticity and are able to respond rapidly to diverse environmental stimuli [7]. In fact, CSCs can switch quickly from glycolysis to oxidative phosphorylation and vice versa, to meet their diverse metabolic needs of ATP production and consumption. However, many recent studies have highlighted the fact that mitochondrial biogenesis plays an especially vital role in CSCs [8–12]. Therefore, halting mitochondrial biogenesis or respiration, may be a vulnerability that we can exploit for their more effective eradication.

Interestingly, natural products and dietary supplements may also show anti-cancer properties. Indeed, we previously investigated the effect of Matcha green tea on proliferation and metabolism in MCF7. Moreover, we used proteomics analysis to dissect how this dietary supplement was able to alter glycolytic and mitochondrial pathways, as well as others related to stem cells and DNA damage/repair [13]. In addition, we also studied how vitamin C [14, 15], bergamot [16] and berberine [17] affect the proliferation of CSCs.

In the current study, we investigated the potential therapeutic effects of several classes of compounds (dietary supplements, FDA-approved drugs, natural products, flavours, vitamins), by assessing their ability to halt the propagation of breast cancer stem cells, using the MCF7 cell line as a model system.

Finally, we focused on the metabolic effects in MCF7 cells of the most promising compounds, two dietary supplements, quercetin and glucosamine, and of the FDA-approved drug, carvedilol. We used the Seahorse XFe96 Analyzer to measure the oxygen consumption rate (OCR) and the glycolysis (ECAR). Our results show that these three compounds can significantly interfere with cancer cell metabolism, resulting in the suppression of CSC propagation. Therefore, we believe that these compounds should be investigated further.

## MATERIALS AND METHODS

### Materials

MCF7 were purchased from ATCC. Cells were cultured in media DMEM (D6546, Sigma-Aldrich). Cell culture media (DMEM/F12) for mammosphere assays was purchased from Life Technologies. Sulforhodamine B (SRB), 1x Trypsin-EDTA, 2-hydroxyethylmethacrylate (poly-HEMA) were purchased from Sigma-Aldrich, as well as the beta-blocker carvedilol. Quercetin, glucosamine hydrochloride, ciprofloxacin, tannic acid, chlorophyllin sodium copper salt, azelaic acid, adipic acid, citral, limonene, nicotinamide and nicotinic acid were from SLS Scientific Laboratory Supplies Ltd. Finally, aloin and aloe emodin were purchased from Santa Cruz Biotechnology.

### Mammosphere assay

From adherent MCF7 cells, we prepared a single cell suspension using enzymatic (1x Trypsin-EDTA) and manual disaggregation (25-gauge needle) [18]. Three thousand cells were plated into mammosphere medium (DMEM-F12/B27/20ng/ml EGF/PenStrep), under non-adherent conditions, in 6-wells plates coated with poly-HEMA. We counted the number of 3D spheroids with a diameter >50 μm, after five days of culture. All experiments were performed in triplicate and repeated three times independently.

### Seahorse analysis

To evaluate the extracellular acidification rates (ECAR) and the oxygen consumption rates (OCR), we used the Seahorse XF96 metabolic flux analyser (Agilent Technologies, Inc.). Fifteen thousand MCF7 cells were seeded per well, into XF96-well cell plates, and cultured at 37° C in an incubator with a 5% CO2 humidified atmosphere. MCF7 cells were cultured in DMEM supplemented with 10% FBS (Foetal Bovine Serum), 2 mM GlutaMAX, and 1% Pen- Strep. After twenty-four hours from plating, the cells were incubated in the presence or absence of quercetin, glucosamine hydrochloride or carvedilol. After forty-eight hours, cells were washed in pre-warmed XF assay media, as previously described [19]. ECAR and OCR measurements were normalized by cellular protein content (SRB). Data sets were analysed using XFe-96 software and Excel, then Student’s t-test calculations were performed. All experiments were performed in sextuplicate and repeated three times independently.

### SRB assay

SRB is a colorimetric assay for cytotoxicity, based on the measurement of cellular protein content. Briefly, MCF7 cells in monolayers were first fixed with 10% trichloroacetic acid and then washed with 1% acetic acid after incubation with SRB. The dye dissolved in 10 mM Tris base solution, and the OD determined at 565 nm, using a microplate reader [20].

### Statistical analysis

All data are presented as the means ± SEM. The Student’s t-test was used to determine significance. p < 0.05 was considered statistically significant. * p < 0.05, ** p < 0.01, *** p < 0.001, ****p < 0.0001.

## RESULTS

### Compound screening

Here, our goal was to identify key compounds that effectively inhibit the anchorage-independent growth and propagation of cancer stem cells (CSCs), using MCF7 cells as a model system. Briefly, these compounds can be classified within 5 sub-categories: 1) dietary supplements; 2) FDA-approved drugs; 3) natural products; 4) flavours; and 5) vitamins. See Table 1.

**Table 1:** Compounds tested for inhibition of MCF7 cancer stem cells (CSC) propagation.

**Compounds**	**~IC-50**
**Dietary Supplements**	
Quercetin	20-40 μM
Glucosamine	5 mM (4X more potent than 2-DG)
**FDA-approved Drugs**	
Carvedilol (beta-blocker)	25 μM
Ciprofloxacin (antibiotic)	100 μM
**Natural Products**	
Aloe Emodin	10-15 μM
Aloin	< 50 μM
Tannic Acid	25 μM
Chlorophyllin Sodium Copper Salt	50-100 μM
Azelaic Acid	5-10 mM
Adipic Acid	5-10 mM
**Flavours**	
Citral	10-50 μM
Limonene	> 50 μM
**Vitamins**	
Nicotinamide (precursor of NADH)	Increases Stemness (10-20 μM)
Nicotinic Acid (Niacin; Vit B3)	No effect

List of compounds and relative IC50. Screening was based on their effects on CSCs propagation, using the mammosphere formation assay. Compounds are divided in five categories: dietary supplements, FDA-approved drugs, natural products, flavours and vitamins.

To assess their potential effect(s) on cancer stem cell activity, we cultured MCF7 cells under low-attachment conditions, in the presence or absence of a given compound. We evaluated CSC activity after five days of culture, by counting the number of mammospheres formed.

We first analysed CSC propagation after treatment with two dietary supplements: quercetin and glucosamine. Quercetin is a flavonoid present in vegetables, fruits and beverages. It has been extensively studied as a chemo-prevention agent in several cancer models [21–23]. It has anti-oxidant, anti-inflammatory and anti-cancer activities [24–30]. Glucosamine is a monosaccharide, precursor used for the glycosylation of proteins and lipids. It is naturally present, for example, in animal bones, bone marrow and the shells of shellfish.

**Figure 1:**
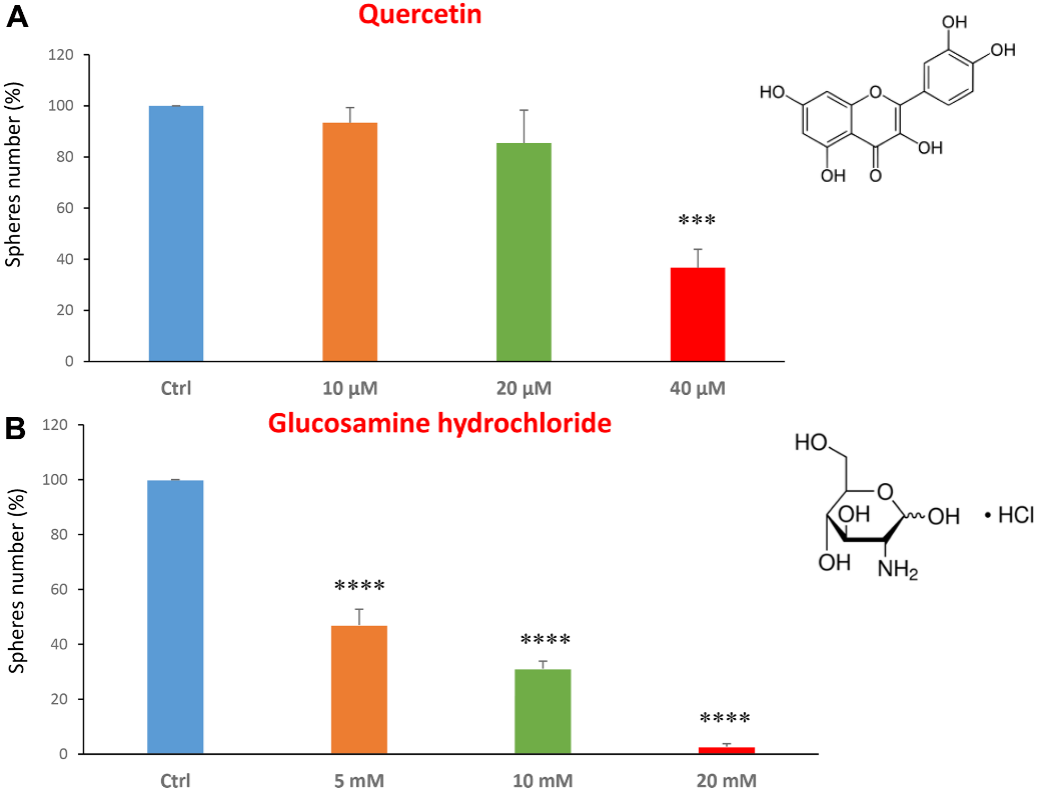
**Dietary supplements decrease CSC propagation.** The effects of two dietary supplements, quercetin and glucosamine hydrochloride, are shown. (**A**) Note that quercetin is effective in inhibiting CSC propagation, at a concentration of 40 μM and its IC50 falls in the range of 20 and 40 μM concentration. (**B**) Note that glucosamine significantly decreases mammosphere number, at concentrations of 5, 10 and 20 mM. Bar graphs are shown as the mean ± SEM; t-test, two-tailed test. ***p < 0.001, ****p < 0.0001. Chemical formulae are indicated.

We tested the quercetin at concentrations of 10, 20 and 40 μM. Figure 1A shows that at the concentration of 40 μM, quercetin was effective in halting CSC propagation by over 60%, and its IC50 fell in the range between 20 and 40 μM. In Figure 1B, results with glucosamine are shown, over the range of 5 to 20 mM. Note that the lowest concentration tested is already effective as an inhibitor of CSC propagation. Interestingly, glucosamine (2-amino-2-deoxy-D-glucose) is structurally related to another well-established metabolic inhibitor, namely 2-DG (2-deoxy-D-glucose). Based on our previous studies using 2-DG in the same MCF7 CSC assay [14, 31], glucosamine appears to be approximately 4 times as potent.

Next, we investigated the effects of two FDA-approved drugs: the beta-blocker carvedilol and the antibiotic ciprofloxacin. Carvedilol, brand name Coreg, is a beta-blocker and is used to treat mild to severe congestive heart failure [32, 33].

**Figure 2:**
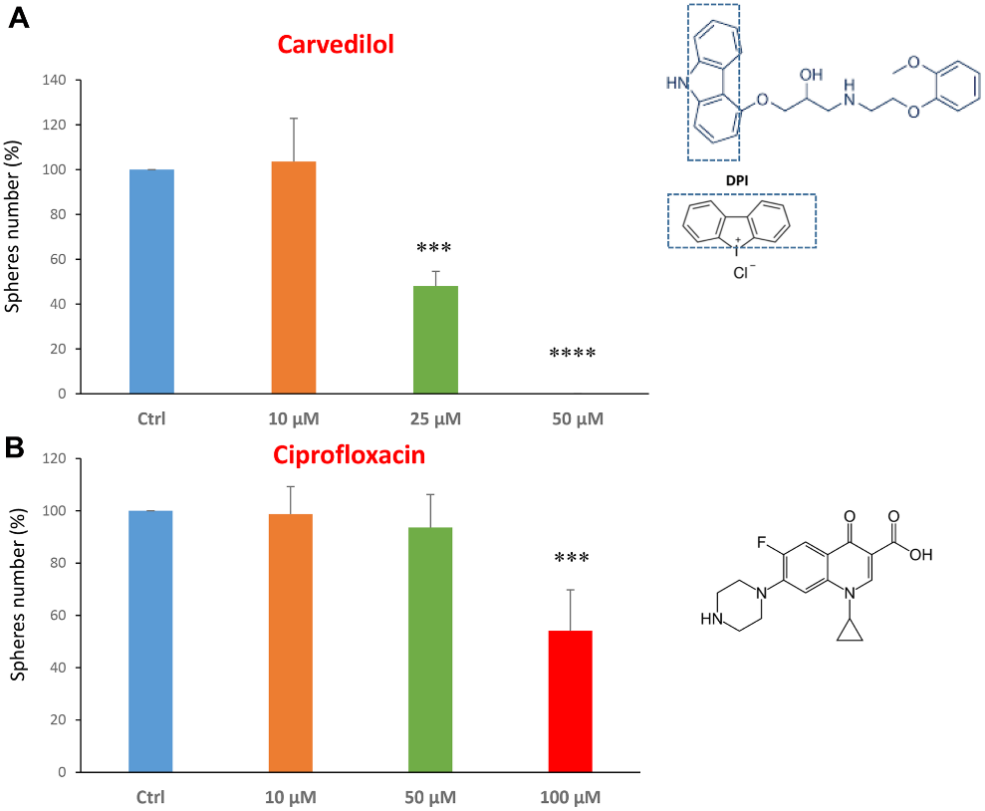
**FDA-approved drugs decrease mammosphere formation.** The effects of two FDA-approved drugs, carvedilol and ciprofloxacin, are shown. (**A**) Note that carvedilol is effective in inhibiting CSC propagation, at a concentration of 25 μM, its IC50, and 50 μM completely inhibits mammosphere formation. (**B**) Ciprofloxacin significantly decreases mammosphere number, at the concentrations of 100 μM, its IC50. Bar graphs are shown as the mean ± SEM; t-test, two-tailed test. ***p < 0.001, ****p < 0.0001. Chemical formulae are indicated.

We tested carvedilol at the concentrations of 10, 25 and 50 μM. The IC50 was 25 μM and the highest dose was so potent as to completely block the mammosphere formation (Figure 2A). However, ciprofloxacin was less potent, with an IC50 of approximately 100 μM (Figure 2B).

Using this approach, we also focused on compounds that are found naturally in plants, or in vegetables and as additive in certain foods. Firstly, we tested two compounds related to each other, aloin emodin and the aloin. These are distinguished only by the fact that aloin emodin lacks a sugar compared to aloin.

**Figure 3:**
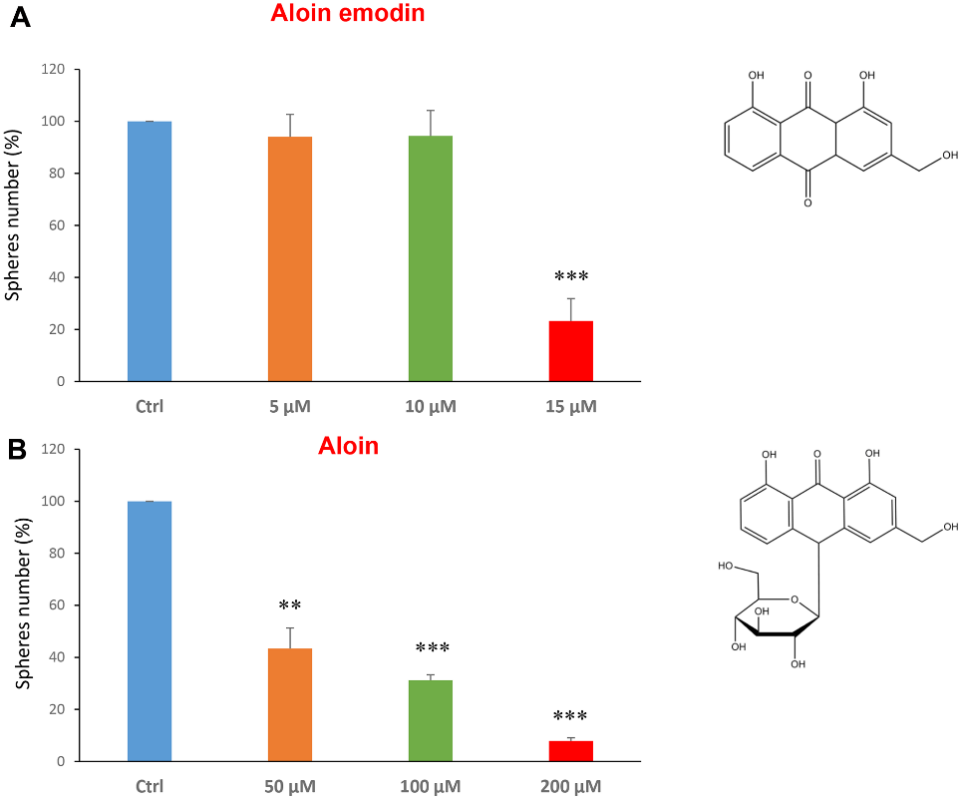
**Natural products derived from plant aloe latex decrease mammosphere formation.** The effects of two natural products, aloe emodin and aloin, are shown. (**A**) Aloe emodin is a compound, with similar biological characteristics of aloin, but lacking a sugar moiety. Note that aloe emodin is effective in inhibiting CSC propagation, by >75% at a concentration of 15 μM. Its IC50 is between 10-25 μM. (**B**) Aloin or barbaloin significantly decreases mammosphere number at a concentration of 50 μM, its IC50. At 200 μM, it reduces the sphere formation by > 90%. Bar graphs are shown as the mean ± SEM; t-test, two-tailed test. **p < 0.01, ***p < 0.001. Chemical formulae are indicated.

Aloin (or barbaloin) is a natural anthraquinone extracted from the plant aloe latex and together with aloe emodin, that lacks a sugar group compared to the first, is widely used as an anti-inflammatory and shows anti-cancer activity [34]. Figure 3A shows that aloin emodin at the concentration of 15 μM was effective in reducing CSC propagation by over 70%. Aloin was also effective at all three concentrations tested of 50, 100 and 200 μM (Figure 3B).

**Figure 4:**
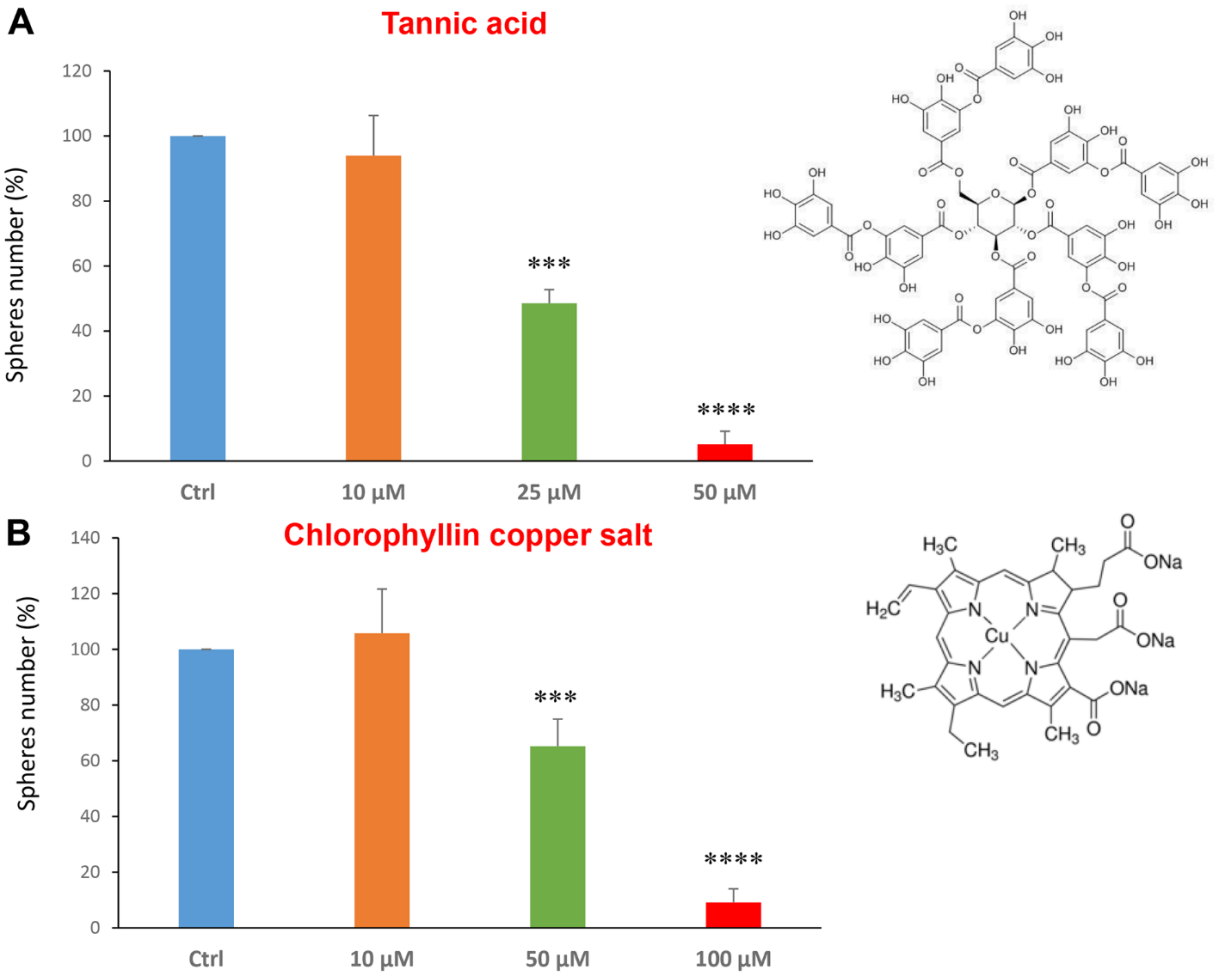
**Natural products, tannic acid and chlorophyllin, were able to decrease mammosphere formation.** We tested the effects of more natural compounds, such as tannic acid and chlorophyllin copper salt. (**A**) Tannic acid is a type of polyphenol. Interestingly, it is effective in inhibiting CSC propagation, at concentrations >10 μM; its IC50 is 25 μM. (**B**) Chlorophyllin is a derivative of chlorophyll which significantly decreases the mammosphere number starting at a concentration of 50 μM and reduces propagation by > 90% at a concentration of 100 μM. Bar graphs are shown as the mean ± SEM; t-test, two-tailed test. ***p < 0.001, ****p < 0.0001. Chemical formulae are indicated.

Tannic acid is a polyphenol, a specific form of tannin, naturally found in the nutgalls made by insects on twigs of oak trees. It has been also used as embalming material of mummies in ancient Egypt [35, 36]. Tannic acid is a potent anti-oxidant with anti-proliferative effects on diverse types of cancer [37]. Tannic acid was tested at the concentrations of 10, 25 and 50 μM, revealing an IC50 of approximately 25 μM (Figure 4A).

Chlorophyll is present in green leaves of vegetables as spinach and is a food colouring agent. It has been shown exhibit anti-oxidant and anti-apoptotic effects [38, 39]. Figure 4B shows the results obtained with chlorophyllin, which has its IC50 in the range between 50 and 100 μM.

**Figure 5:**
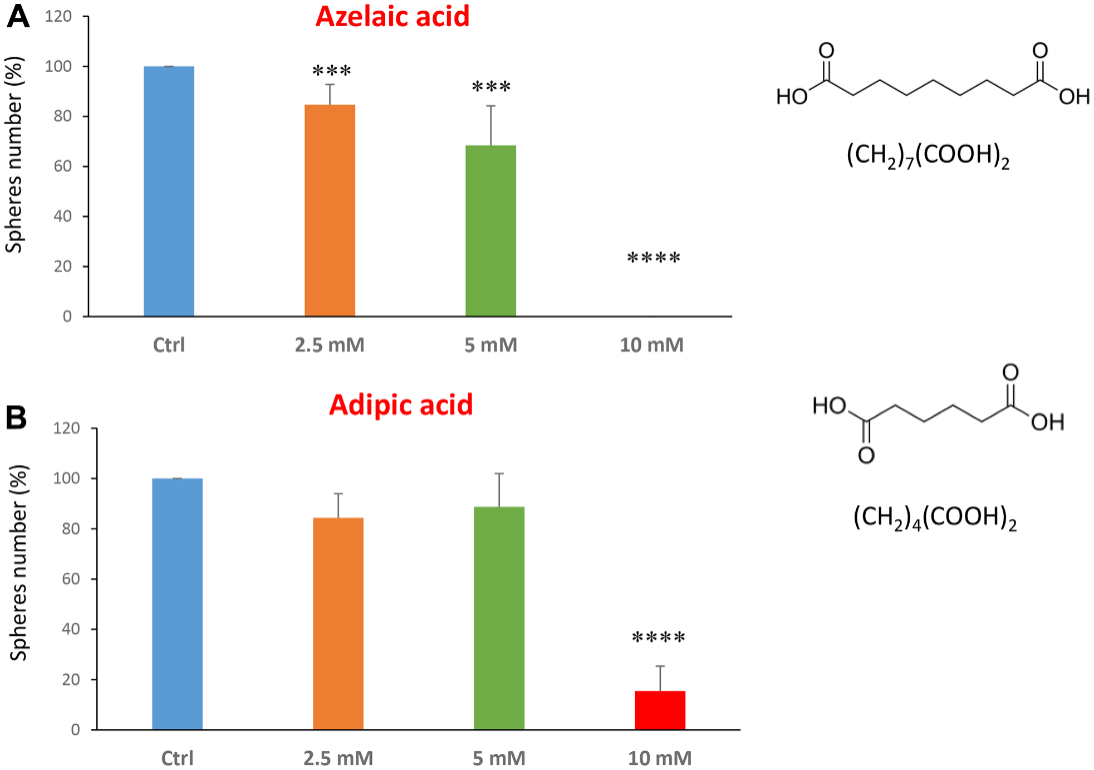
**Natural products, azelaic and adipic acids, decrease mammosphere formation.** Finally, we tested the effects of two more natural compounds, such as azelaic acid and adipic acid. (**A**) Azelaic acid is a saturated dicarboxylic acid and it is effective in inhibiting CSC propagation, starting at a concentration of 2.5 mM, with complete inhibition at a concentration of 10 mM. (**B**) Adipic acid is another dicarboxylic acid that significantly blocks CSC propagation, with near complete inhibition at 10 mM, similarly to azelaic acid. Bar graphs are shown as the mean ± SEM; t-test, two-tailed test. ***p < 0.001, ****p < 0.0001. Chemical formulae are indicated.

The last two compounds in this category were azelaic acid and adipic acid (Figure 5A, 5B). Azelaic acid is found in wheat, rye and barley. It inhibits mitochondrial enzymes of the respiratory chain and enzymes involved in DNA synthesis showing antiproliferative and cytotoxic effects in melanoma, bladder and breast cancers, and leukaemia [40–42]. Adipic acid is used mainly in the production of nylon and is also used as a food additive [43, 44]. Azelaic acid displayed effectiveness starting at a concentration of 2.5 mM, with an IC50 between 5 and 10 mM; note that at 10 mM the propagation of CSCs was completely halted. Similarly, adipic acid also showed promising inhibitory effects.

**Figure 6:**
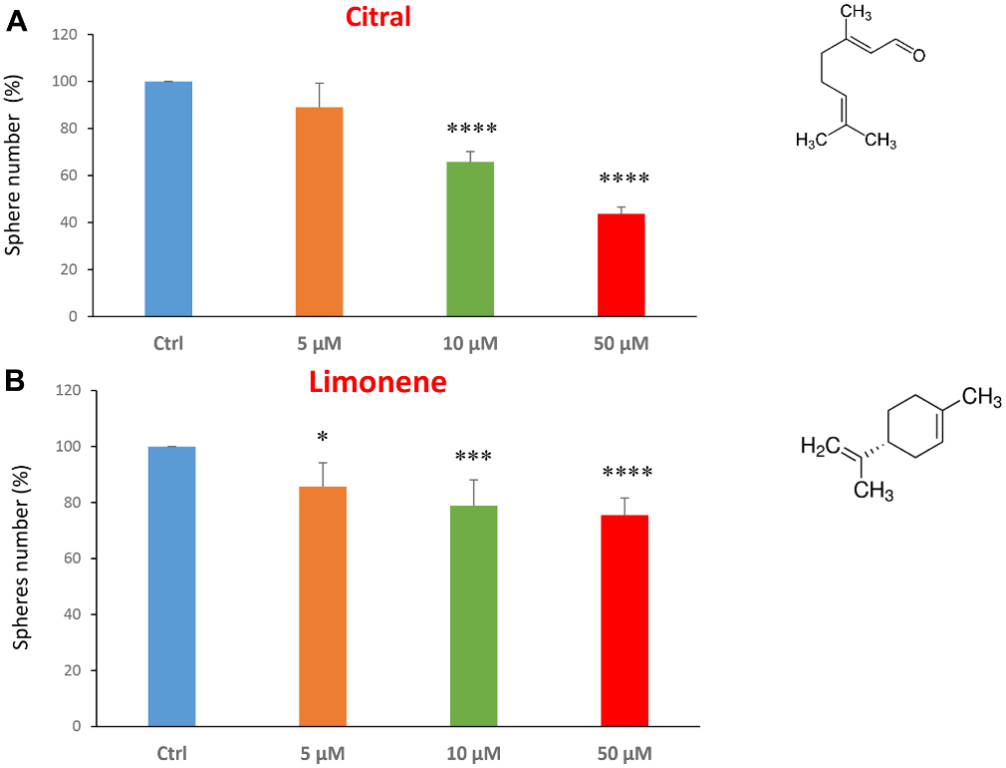
**Flavours, citral and limonene, decrease mammosphere formation.** Next, we tested the effects of two flavours, such as citral and limonene. (**A**) Citral or lemonal is effective in inhibiting CSC propagation, starting at the concentration of 10 μM, with an IC50 near 50 μM. (**B**) Limonene is a flavouring that significantly decreases the mammosphere formation, but was less effective than the closely related molecule, Citral. Bar graphs are shown as the mean ± SEM; t-test, two-tailed test. *p < 0.05, ***p < 0.001, ****p < 0.0001. Chemical formulae are indicated.

We next investigated flavour-related compounds, such as citral and limonene (Figure 6A, 6B). Citral (or lemonal) is naturally present in lemons, oranges and limes. Limonene is used as a flavouring in foods, beverages and chewing gum. Citral had an IC50 between 10-50 μM and limonene greater than 50 μM.

**Figure 7:**
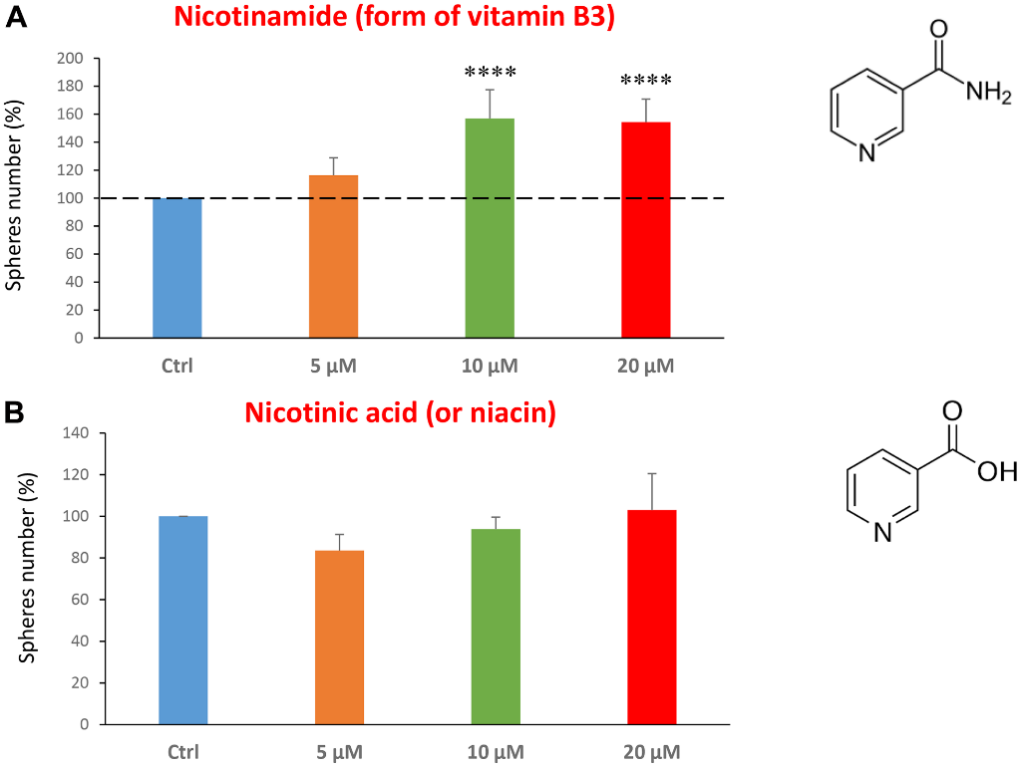
**Testing the efficacy of two forms of vitamin B3 on CSC propagation.** (**A**) Nicotinamide, also known as niacinamide, significantly increases CSC propagation by >1.5-fold, at concentrations of 10 and 20 μM. (**B**) However, Nicotinic acid (or niacin) does not have any effect on mammosphere formation. Bar graphs are shown as the mean ± SEM; t-test, two-tailed test. ****p < 0.0001. Chemical formulae are indicated.

Finally, we assessed the effects of two common vitamins on CSCs proliferation: nicotinamide, which is the active form of vitamin B3, and nicotinic acid (a.k.a, niacin). Nicotinamide is an amide form of vitamin B3, and is found in foods like fish, poultry, eggs and is used as a dietary supplement/medication, to prevent and treat pellagra [45]. Nicotinic acid or niacin is the vitamin B3 and is used to reduce elevated levels of cholesterol [46]. Importantly, nicotinamide and nicotinic acid are both precursors of the co-enzymes nicotinamide adenine dinucleotide (NADH) and nicotinamide adenine dinucleotide phosphate (NADPH) [14]. Interestingly, treatment with nicotinamide significantly increased the CSC propagation, at concentrations of 10 and 20 μM. However, nicotinic acid did not show any significant effects at the doses tested (5, 10, or 20 μM) (Figure 7A, 7B).

### Metabolic validation via seahorse analysis

**Figure 8:**
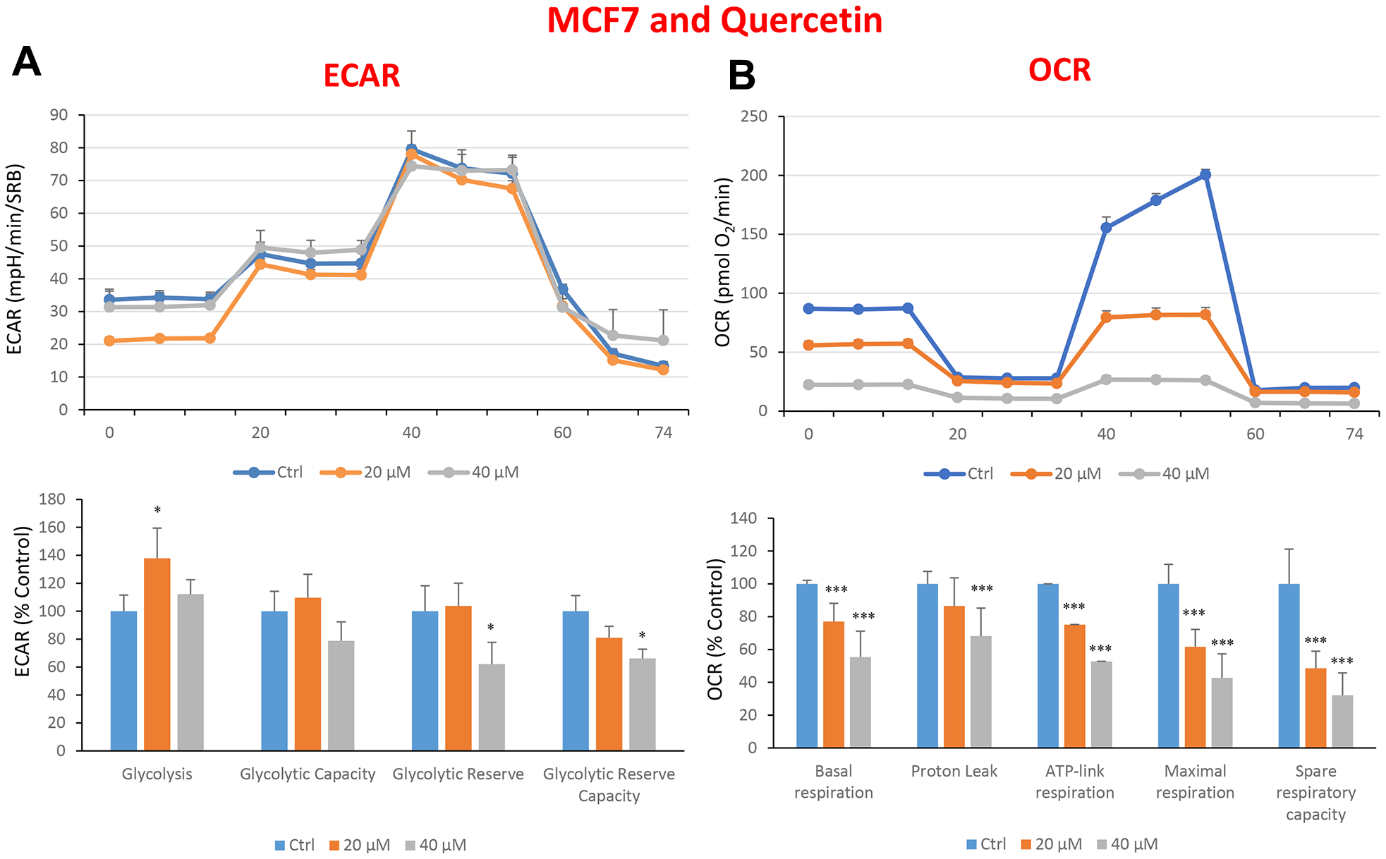
**Treatment with quercetin preferentially reduces mitochondrial oxygen consumption rates in MCF7 cells.** Cells were seeded and treated with quercetin, as described above. Briefly, cells were seeded at a density of fifteen thousand in a 96-well format. (**A**) Extracellular consumption rate (ECAR) was assessed by Seahorse metabolic flux analysis. A representative trace is shown in the top panel. Importantly, quercetin treatment only had minor effects on glycolysis. (**B**) Oxygen consumption rate (OCR) was measured by Seahorse metabolic flux analysis. A representative trace, in the top panel, shows decreased OCR in samples treated with quercetin (20 and 40 μM), versus the vehicle alone control cells. The bar graph (lower panel) shows that quercetin treatment significantly decreases the basal respiration, ATP production, maximal and spare respiration, as compared to the control cells. In panels **A** and **B**, experiments were performed three times independently, with six repeats for each replicate. Bar graphs are shown as the mean ± SEM, t-test, two-tailed test. *p < 0.05, ***p < 0.001.

**Figure 9:**
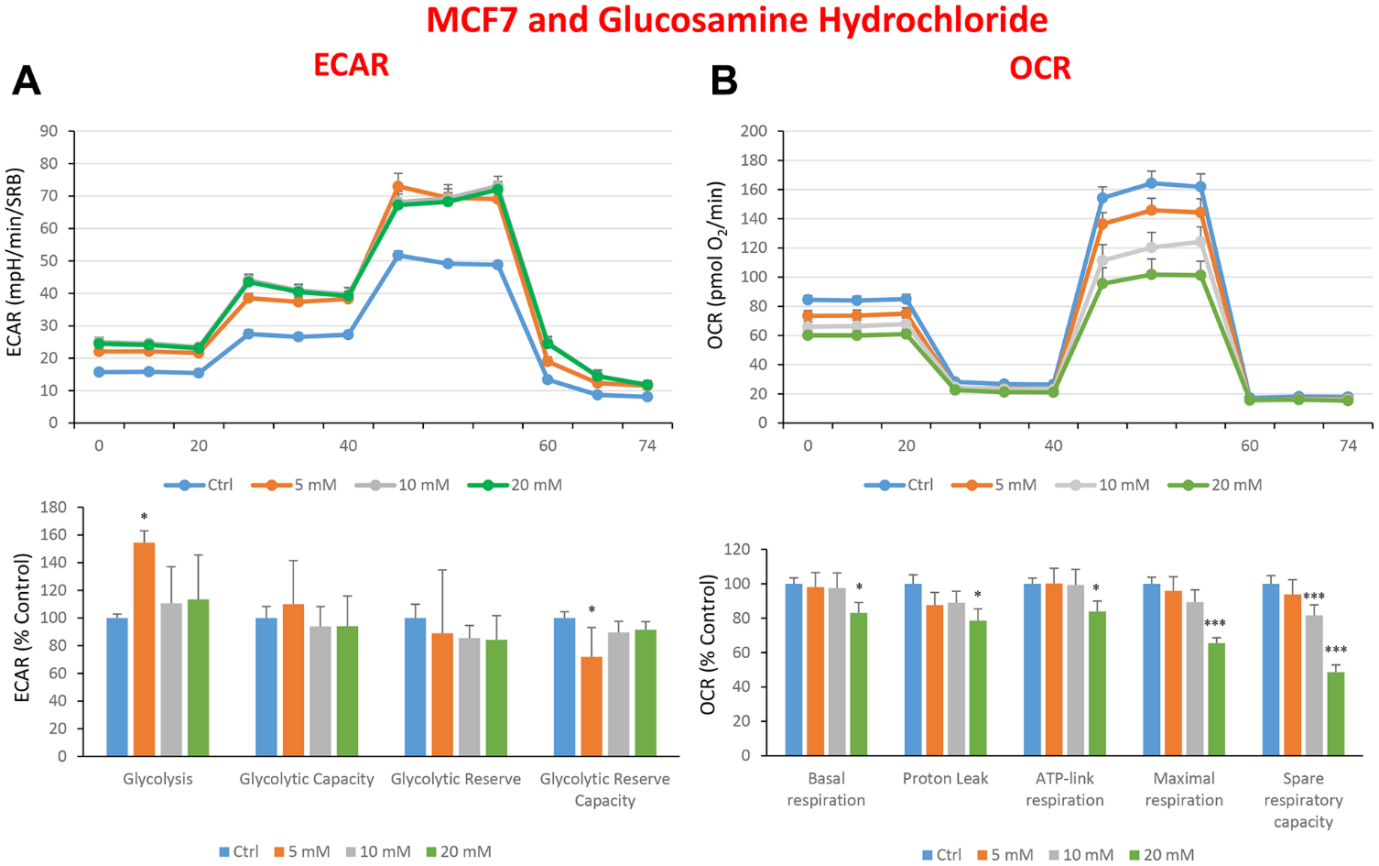
**Treatment with glucosamine hydrochloride reduces mitochondrial oxygen consumption rates in MCF7 cells.** Cells were seeded and treated with glucosamine, as described above. Briefly, cells were seeded at a density of fifteen thousand in a 96-well format. (**A**) Extracellular consumption rate (ECAR) was assessed by Seahorse metabolic flux analysis. A representative trace is shown in the top panel. Importantly, glucosamine treatment only had minor effects on glycolysis. (**B**) Oxygen consumption rate (OCR) was measured by Seahorse metabolic flux analysis. A representative trace, in the top panel, shows decreased OCR in samples treated with glucosamine (20 mM), versus the vehicle alone control cells. The bar graph (lower panel) shows that glucosamine treatment significantly decreases the basal respiration, ATP production, maximal and spare respiration, as compared to the control cells. In panels **A** and **B**, experiments were performed three times independently, with six repeats for each replicate. Bar graphs are shown as the mean ± SEM, t-test, two-tailed test. *p < 0.05, ***p < 0.001.

**Figure 10:**
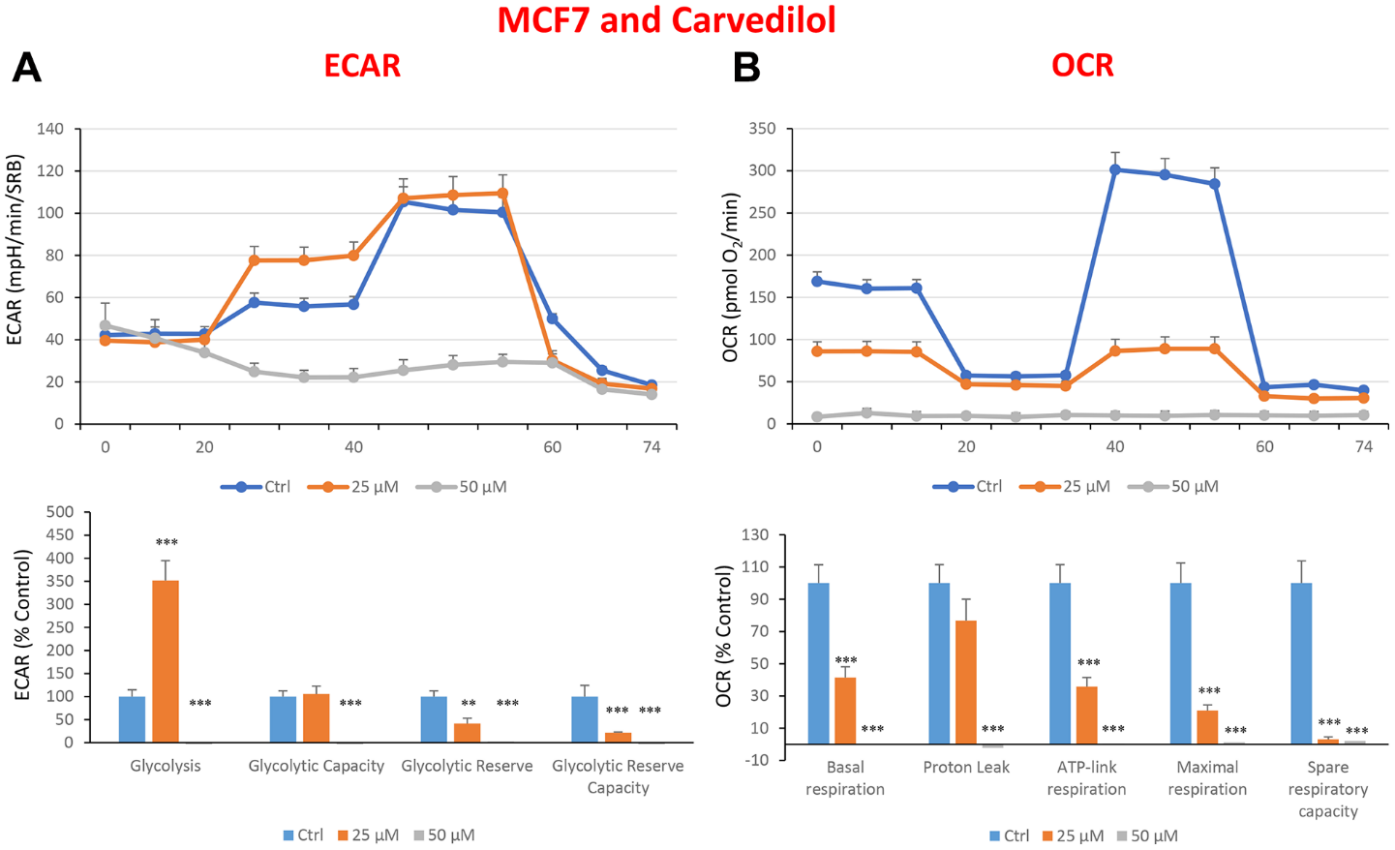
**Treatment with carvedilol differentially affects both glycolysis and oxygen consumption rates in MCF7 cells, in a concentration-dependent manner.** Cells were seeded and treated with carvedilol, as described above. Briefly, cells were seeded at a density of fifteen thousand in a 96-well format. (**A**) Extracellular consumption rate (ECAR) was assessed by Seahorse metabolic flux analysis. A representative trace is shown in the top panel. Importantly, carvedilol treatment induced glycolysis by >3.5-fold at 25 μM, but showed dramatic inhibition of glycolysis at 50 μM. (**B**) Oxygen consumption rate (OCR) was measured by Seahorse metabolic flux analysis. A representative trace, in the top panel, shows progressive decreases in OCR in samples treated with carvedilol (25 and 50 μM), versus the vehicle alone control cells. The bar graph (lower panel) shows that carvedilol treatment significantly decreases the basal respiration, ATP production, maximal and spare respiration, as compared to the control cells. In summary, at 25 μM, carvedilol enhanced glycolysis, but inhibited mitochondrial oxygen consumption. In contrast, at 50 μM, carvedilol inhibited both glycolysis and mitochondrial oxygen consumption. In panels **A** and **B**, experiments were performed three times independently, with six repeats for each replicate. Bar graphs are shown as the mean ± SEM, t-test, two-tailed test. *p < 0.05, ***p < 0.001.

In the literature, it is well documented that propagation of CSCs depends on several factors including an increased mitochondrial metabolism and biogenesis [7, 47, 48]. In this regard, to address an effect of quercetin, glucosamine and carvedilol on cellular metabolic features, we performed analysis with the Seahorse XF Analyzer after a 48-hours treatment with the compound. We measured oxygen consumption rate (OCR) and extracellular acidification rate (ECAR). Interestingly, quercetin significantly increased the glycolysis at the concentration of 20 μM and reduced the glycolytic reserve and glycolytic reserve capacity (maximal capacity of the cells to respond to a higher ATP demand) at the concentration of 40 μM as compare to the untreated control cells (Figure 8A). Moreover, all the OCR parameters were significantly decreased: basal respiration, proton leak, ATP production, maximal respiration and spare respiratory capacity (Figure 8B). Next, we investigated the effect of glucosamine finding that it was able to significantly increase glycolysis at the concentration of 5 mM and decrease the glycolytic reserve capacity (Figure 9A). Importantly, at 20 mM all OCR parameters were significantly decreased but the spare respiratory capacity which was already negatively affected at the dose of 10 mM (Figure 9B). Lastly, we examined carvedilol to see if it could affect cellular metabolism. We treated MCF7 cells with 25 and 50 μM of carvedilol. Results in Figure 10A show that this drug dramatically negatively affected all ECAR parameters, at the maximal concentration of 50 μM. At the dose of 25 μM only glycolytic reserve and glycolytic reserve capacity were significantly decreased. On the contrary, glycolysis was increased more than three times perhaps as an attempt to compensate for the dramatic decrease in all the parameters related to the oxygen consumption rate. Indeed, OCR analysis highlighted that carvedilol had a powerful capability in decreasing the mitochondrial respiration (Figure 10B).

**Figure 11:**
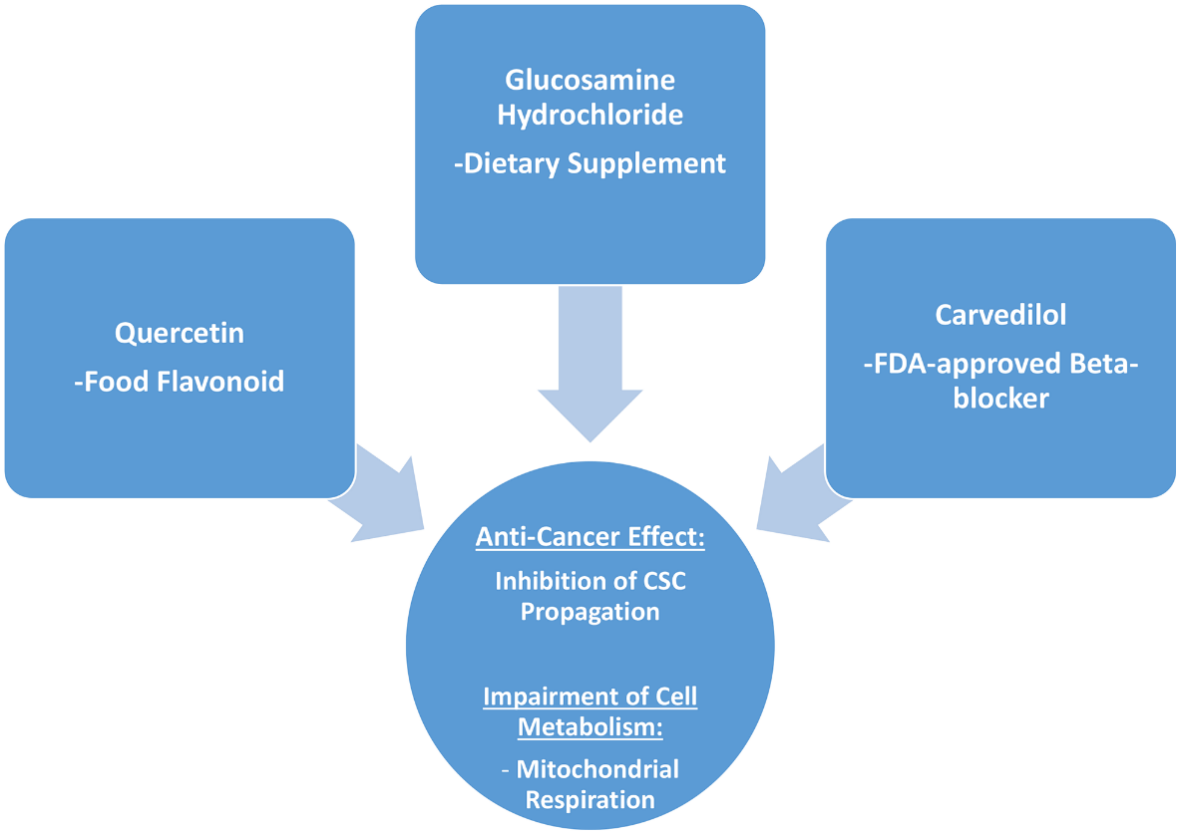
**Summary: Identification of natural products and FDA-approved drugs for targeting cancer stem cell (CSC) propagation.** This scheme summarizes our current results related to quercetin, glucosamine hydrochloride and carvedilol compounds and their effects on i) CSC propagation and ii) energy metabolism in MCF7 cells. Quercetin is flavonoid found in many foods, glucosamine is a dietary supplement, and carvedilol is an FDA-approved beta-blocker. Intriguingly, although these three compounds are so different in their chemical structure, they share the ability to interfere with mitochondrial metabolism and block the propagation of CSCs.

Interestingly, in the end what stands out from this more detailed analysis conducted on quercetin, glucosamine and carvedilol, is that what these three compounds have in common is their effects on mitochondrial respiration, and that they are effective in inhibiting the propagation of MCF7 cancer stem-like cells (Figure 11).

## DISCUSSION

The eradication of cancer stem cells remains a focal point in the battle against cancer, regardless of the type of cancer. Cancer stem cells are considered to be responsible for the dissemination and formation of distant metastases [49], as well as resistance to anti-cancer therapies [50–52]. For this reason, it is vital to find the Achilles’ heel of CSCs. Recently, many investigators have highlighted the importance of the metabolic micro-environment, as well as the metabolic features of CSCs [47, 53–56].

In this study, we investigated the effectiveness of different compounds in decreasing or blocking the anchorage-independent growth of MCF7 cancer stem cells, by using the mammosphere assay, as a rapid *in vitro* screening tool that exploits the ability of CSCs to grow under low-attachment conditions.

Here, we chose to examine the activity of different compounds, which can be classified into 5 different groups: 1) dietary supplements (quercetin and glucosamine); 2) FDA-approved drugs (carvedilol and ciprofloxacin); 3) natural products (aloe emodin, aloin, tannic acid, chlorophyllin copper salt, azelaic acid and adipic acid); 4) flavours (citral and limonene); and 5) vitamins (nicotinamide and nicotinic acid). Our results are summarized schematically in Figure 11.

Glucosamine is widely used in the medical field in the treatment of osteoarthritis [57], and it is well known that it does not have side effects in humans. Moreover, it has been reported as an attractive candidate in lung carcinogenesis, decreasing the lung cancer risk [58]. Interestingly, glucosamine has inhibitory effects on glycolysis [59, 60] and drives general cell ATP depletion [61]. Moreover, it has been also found that glucosamine induced dysfunction of mitochondria as well as that of the peroxisome in human chondrocytes [62]. In our study, the results showed that glucosamine was able to reduce the mammospheres formation efficiency starting from the lowest tested concentration of 5 mM (IC50). This result is relevant because compared to 2-DG (IC50, 20 mM), glucosamine is a more potent glycolytic inhibitor. This evidence adds to the advantage that glucosamine is already used in the medical field, while the 2-DG cannot be administered to humans. In addition, we investigated the metabolic effect of glucosamine on MCF7 cells in adhesion, representing the bulk tumour cells, using the Seahorse Analyzer. Importantly, at 20 mM all OCR parameters were significantly decreased, but the spare respiratory capacity was already negatively affected at a dose of 10 mM. As such, glucosamine may have potential as a therapeutic to halt the proliferation of CSCs, as we are starting to understand the importance of metabolic flexibility, as an intervention point, for decreasing tumour recurrence and metastasis [47, 55].

Also, quercetin appears to be effective against the proliferation of cancer cells, for example, in breast tumour, pancreatic and oral squamous cell carcinomas [63–68]. Importantly, quercetin can modulate pathways associated with different mitochondrial processes [69]. Here, we revealed a decrease in the proliferation of CSCs and moreover, by Seahorse analysis, we showed that this treatment was able to negatively affect the glycolytic reserve capacity. Importantly, almost all OCR parameters were significantly decreased, at both of the concentrations of quercetin tested.

Carvedilol or Coreg is a beta-blocker widely used as a cardio protector in cardiac dysfunction [70, 71]. It is also used to prevent chemotherapy-related cardiotoxicity [72, 73], and cardiac mitochondrial oxidative damage [74]. Studies on malignant breast cancer cells have been performed to investigate the ability of carvedilol in inhibiting their proliferation and migration [75, 76]. Here, we wanted to test its effectiveness in halting breast CSC propagation and its possible role in altering their metabolic pathways. Our findings show that the drug was effective in reducing mammospheres formation (IC50 at 25 μM and complete inhibition at 50 μM), and in reducing the glycolysis and the oxidative respiration parameters, as highlighted by the Seahorse analysis.

We also tested another FDA-approved drug such as the antibiotic ciprofloxacin, which has previously reported to effectively block cell proliferation of bladder and melanoma cancer cells [77, 78]. In addition, this antibiotic has shown effectiveness in altering mammalian mitochondrial DNA replication [79]. Here, ciprofloxacin displayed its IC50 of approximately 100 μM in the mammosphere formation assay.

Next, we investigated the mammosphere formation capacity of aloin, which has been reported to be cytotoxic against two human breast cancer cell lines [80]. Further, aloin was able to induce apoptosis in lung cancer cells causing disruption of mitochondrial membrane potential and inducing ROS production [81].

Then, we tested tannic acid, a potent anti-oxidant and anti-proliferative agent that is effective in inhibiting EGFR/STAT signalin, resulting in cell cycle arrest and apoptosis [82]. Chlorophyllin, another natural compound, also has as anti-cancer effects [83, 84]. Interestingly, chlorophyllin inhibits oxidative phosphorylation in rat liver mitochondria [85]. Moreover, we tested the azelaic acid already described to be effective against tumour as well as in cutaneous disorders [40, 86, 87], beside its ability to inhibit mitochondrial respiration and promoting mitochondrial damage [88], and adipic acid which it is used in the industrial production of nylon [44]. Finally, we tested the effectiveness of citral and limonene which are already used in medicine [89]. Citral is well known to have an antifungal activity altering oxidative phosphorylation [90], by altering the mitochondrial membrane potential in MDA-MB-231 cells [91]. In addition, limonene plays a key role in regulation of oxidative stress mediated by ROS in a broad variety of organisms [92].

We have previously shown that the upregulation of NAD+ salvage pathways increases stemness [14], and here we confirm our results since the administration of nicotinamide increased the proliferation of breast CSCs [14]. Very recently, an independent group reported that decreased intracellular NAD, due to the up-regulation of miR-381, was able to induce apoptosis in breast cancer cells [93].

Intriguingly, several of the agents tested here shared the property of interfering with mitochondria and their function.

## Conclusions and Future Directions

**Figure 12:**
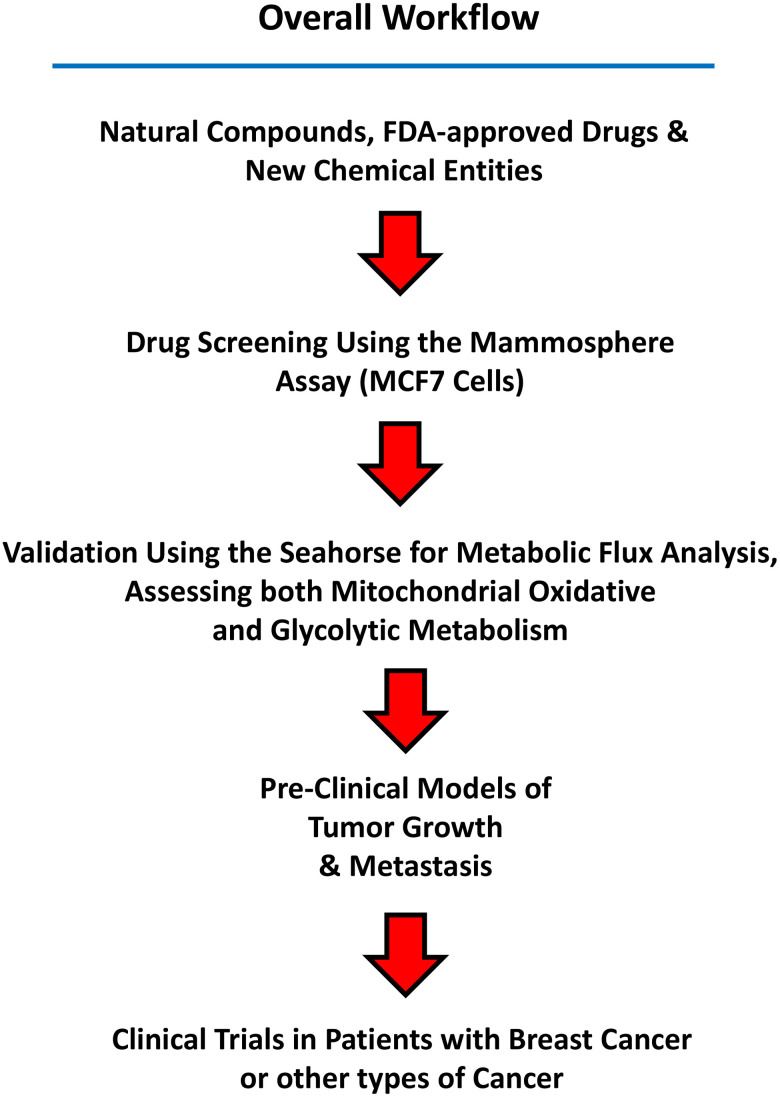
**Summary of the Workflow.** Candidate natural compounds, FDA-approved drugs, and/or new chemical entities are subjected to drug screening, using the 3D mammosphere assay (MCF7 cells). Positive hits are then validated as metabolic inhibitors, by using the Seahorse, to directly measure oxygen consumption and metabolic flux. Small chemical entities showing anti-mitochondrial activity can then be further validated in pre-clinical models of tumor growth and metastasis. Finally, clinical trials in patients with breast cancer (or other cancer types) can be carried out to validate *in vivo* that a given compound eradicates CSCs, using CSC-specific markers, such as CD44 and ALDH1 by immuno-histochemistry.

Here and in previous reports, we have identified numerous chemical entities, with anti-mitochondrial activity, that can target and eradicate CSCs *in vitro*. Figure 12 shows a summary diagram that illustrates the workflow of this experimental screening and clinical strategy. A relatively comprehensive list of these compounds can be found in the following review article [94].

Next steps would include: 1) their evaluation in pre-clinical animal models; and 2) clinical trials, as well. For example, using Doxycycline, we have previously shown that it eradicates CSCs and prevents metastasis in a preclinical animal model [95]. Moreover, a phase II clinical trial (window study) showed that Doxycycline eradicates CSCs *in vivo*, using CD44 and ALDH1 as CSC-markers [96].

Therefore, Doxycycline provides the first example that this strategic approach is indeed successful.
